# Identification of fatty acids synthesis and metabolism-related gene signature and prediction of prognostic model in hepatocellular carcinoma

**DOI:** 10.1186/s12935-024-03306-4

**Published:** 2024-04-07

**Authors:** Ai Zhengdong, Xing Xiaoying, Fu Shuhui, Liang Rui, Tang Zehui, Song Guanbin, Yang Li, Tang Xi, Liu Wanqian

**Affiliations:** 1 Key Laboratory of Biorheological Science and Technology (Chongqing University), Ministry of Education, College of Bioengineering, Chongqing University, 174 Shazheng Street, Chongqing, 400000 People’s Republic of China; 2 Gastrointestinal Cancer Center, Chongqing University Cancer Hospital, Chongqing, 400000 People’s Republic of China

**Keywords:** Fatty acids, Immune microenvironment, Hepatocellular carcinoma, Acetyl-CoA carboxylase1, Cancer stemness

## Abstract

**Background:**

Fatty acids synthesis and metabolism (FASM)-driven lipid mobilization is essential for energy production during nutrient shortages. However, the molecular characteristics, physiological function and clinical prognosis value of FASM-associated gene signatures in hepatocellular carcinoma (HCC) remain elusive.

**Methods:**

The Gene Expression Omnibus database (GEO), the Cancer Genome Atlas (TCGA), and International Cancer Genome Consortium (ICGC) database were utilized to acquire transcriptome data and clinical information of HCC patients. The ConsensusClusterPlus was employed for unsupervised clustering. Subsequently, immune cell infiltration, stemness index and therapeutic response among distinct clusters were decoded. The tumor immune dysfunction and exclusion (TIDE) algorithm was utilized to anticipate the response of patients towards immunotherapy, and the genomics of drug sensitivity in cancer (GDSC) tool was employed to predict their response to antineoplastic medications. Least absolute shrinkage and selection operator (LASSO) regression analysis and protein–protein interaction (PPI) network were employed to construct prognostic model and identity hub gene. Single cell RNA sequencing (scRNA-seq) and CellChat were used to analyze cellular interactions. The hub gene of FASM effect on promoting tumor progression was confirmed through a series of functional experiments.

**Results:**

Twenty-six FASM-related genes showed differential expression in HCC. Based on these FASM-related differential genes, two molecular subtypes were established, including Cluster1 and Cluster2 subtype. Compared with cluster2, Cluster1 subtype exhibited a worse prognosis, higher risk, higher immunosuppressive cells infiltrations, higher immune escape, higher cancer stemness and enhanced treatment-resistant. PPI network identified Acetyl-CoA carboxylase1 (ACACA) as central gene of FASM and predicted a poor prognosis. A strong interaction between cancer stem cells (CSCs) with high expression of ACACA and macrophages through CD74 molecule (CD74) and integrin subunit beta 1 (ITGB1) signaling was identified. Finally, increased ACACA expression was observed in HCC cells and patients, whereas depleted ACACA inhibited the stemness straits and drug resistance of HCC cells.

**Conclusions:**

This study provides a resource for understanding FASM heterogeneity in HCC. Evaluating the FASM patterns can help predict the prognosis and provide new insights into treatment response in HCC patients.

**Supplementary Information:**

The online version contains supplementary material available at 10.1186/s12935-024-03306-4.

## Background

Fatty acids (FAs) are composed of hydrocarbon chains with varying lengths and degrees of desaturation, which are used to synthesize a variety of lipids for the construction of biological membranes, energy metabolism and storage, and signaling molecules [1–3]. FAs are important components of triacylglycerides (TAGs), which can be assembled and stockpiled during enough nutrition and release tremendous energy upon decomposition [4]. Intriguingly, tumor cells often encounter hypo-nutrient conditions, which force them to make adaptive changes to meet their high lipid demands for energy and various biomaterials [5]. For example, tumors convert glucose or acetate into lipids at a comparatively higher rate, however this process is still too slow to meet the lipid requirements of infinitely replicating tumors [6]. Recent study verifies that cellular FAs of almost tumor cells are de novo synthesis to support their own lipid requirements [7]. Notably, the metabolic dysfunction of FAs has been increasingly reorganized to selectively prime the ferroptosis of tumors, stimulate macrophage functions, control regulatory T cell differentiation and autoimmunity, and influence tumor progression [8–11]. All these studies suggest that FAs biosynthesis and metabolism (FASM) are crucial for cancer cell growth and survival. Nevertheless, understanding tissue-specific FASM is an important foundation for understanding cancer progression and malignancy.

Hepatocellular carcinoma (HCC), with increasing incidence, is a ubiquitous form of liver cancer [12]. Attractively, liver is main site of de novo FA biosynthesis [13]. The prevalence of FA flux-driven liver inflammation, fibrosis, cirrhosis and eventual HCC is showing a rapidly increasing trend [14, 15], indicating that FA synthesis is closely bound up with the progression of HCC. In HCC tumorigenesis, ubiquitin-specific protease 22 (USP22) directly mediates the deubiquitylation and stabilization of peroxisome proliferator-activated receptor gamma (PPARγ). In turn, the stabilization of PPARγ facilitates the expressions of acetyl-CoA carboxylase (ACC) and ATP citrate lyase (ACLY) to promote the de novo FAs synthesis [16]. Moreover, hepatocyte-specific FAs metabolic reprogramming is a momentous symbol of liver carcinogenesis and development [17]. For instance, the NADPH oxidase 4 (NOX4) deletion promotes HCC progression by reprogramming FAs metabolism in a NRF2/MYC-dependent manner [18]. Aberrant RNA modifications result in the dysregulated translation level of mRNAs involved in FAs metabolism and HCC development [19]. Furthermore, altered energy metabolism of tumor cells drives the immune cell response in tumor microenvironment that accelerates tumor progression. Recent study show that Piwi Like RNA-Mediated Gene Silencing 1 (PIWIL1) increases oxygen utilization and energy production via FAs metabolism and attracts myeloid-derived suppressor cells (MDSCs) into the tumor microenvironment, generating tumor immune suppression in HCC [20]. Whereas, clinical effectiveness of above finds is restricted in predicting cancer progression and treatment response, and most of them have not been confirmed with a large number of clinical samples.

The depth and accuracy of omics analysis can be improved by integrating multi-omics data and constructing computational models [20]. Several analysis methods based on omics data have been used to identify biomarkers during the development of metabolic liver disease [21]. In our work, we utilized a gene signature of FASM to distinguish patients with different FASM patterns. We then performed a comprehensive analysis to assess differences in risk models, immune cell infiltration characteristics and tumor stemness features between the FASM patterns. In addition, the PPI networks and scRNA-seq were used to analyze the expression heterogeneity of hub gene ACACA and intercellular communication in HCC cell subtypes. Finally, we validated the important role of ACACA in HCC though multi-layered expression verification and a battery of in vitro functional exploration. Our work highlights the key role of FASM in HCC progression by identifying their molecular characteristics, physiologic function and clinic prognosis, holding promise for therapeutic strategies targeting FASM pathways.

## Methods

### Gene set and raw data

A total of 664 datasets of HCC samples were supplied by two public databases. RNA sequencing data underwent variance-stabilizing transformation (VST) using the DESeq2 package in R. These datasets included 424 samples were from TCGA-LIHC cohort (https://portal.gdc.cancer.gov/) [21] and 240 samples were from ICGC-LIRI-JP cohort (https://dcc.icgc.org/) [22]. Importantly, the TPM matrix normalized by counts in TCGA and ICGC was used for subsequent analysis. And the “log2” was used to reduce the TPM matrix variability to make the TPM matrix closer to the normal distribution. The clinical information of samples such as age, gender, race, treatment type, stage, and status were also obtained from TCGA and ICGC (Additional file 2: Table S1 and Additional file 3: Table S2). Additionally, the single cell RNA sequencing dataset of GSE125449 was from the GEO [23]. We established a gene set by obtaining FASM-related genes from the molecular signatures database (MSigDB) database (https://www.gsea-msigdb.org/gsea/msigdb/index.jsp) (Additional file 4: Table S3) [24].

### Integrated omics analysis

To identify differential expression of FASM-related genes between precancerous and cancerous tissues, we utilized the DESeq2 package in R with thresholds for significance set at a false discovery rate < 0.05 and | Log2 fold change |> 1 [25]. Moreover, we identified FASM-associated genes that significantly influenced overall survival (OS) in HCC through univariate Cox regression analysis using the Survival package. The maftools package were employed to describe somatic mutations of these genes in HCC patients [26].

### FASM patterns by unsupervised clustering

For unsupervised clustering, we employed the ConsensusClusterPlus package [27], selecting an optimal number of subtypes based on proportion of ambiguous clustering (PAC). Principal component analysis (PCA) and t-Distributed Stochastic Neighborhood Embedding (tSNE) methods were carried out to compare the expression levels among different FASM subtypes. In order to assess survival outcomes within clusters derived from the TCGA-LIHC and ICGC-LIRI-JP cohorts, survminer and survival packages were used to draw Kaplan–Meier survival curves and conduct log-rank tests.

### Prognostic model construction

Based on 26 FASM associated genes, the reliability factors of LASSO regression analysis were executed by using multivariate Cox regression method [28]. Patients with LIHC were distinguished into high- or low-risk groups according to the polygenic risk score obtained by the prognostic features. In addition, area under curve (AUC) score of the receiver operating characteristic (ROC) curve was used for evaluating the prediction ability of prognostic signatures. The R packages survival, rms and timeROC were performed to establish and verify the prognostic model of FASM associated genes.

### Immune cell infiltration characteristics analysis

We employed single-sample gene set enrichment analysis (ssGSEA) to measure the relative abundance of 28 immune cell types associated with immune response [29, 30]. The ssGSEA algorithm enabled us to express the relative abundance of each immune cell as an enrichment score normalized to a ranging from 0 to 1. This approach was utilized to evaluate the LIHC cohort, and explore the heterogeneity of TME among different FASM subtypes in LIHC. To evaluate the immune cell status in LIHC patients, we utilized CIBERSORT, a deconvolution method that utilizes gene expression profiles [31, 32]. Additionally, we investigated the distribution of 22 immune cell types using CIBERSORT and examined their infiltration levels in high- and low-risk populations. Furthermore, we explored the correlation between risk scores and immunologic function. For simulating tumor immune escape mechanisms and predicting potential response to tumor immunotherapy, we adopted TIDE algorithm [33].

### Gene set variation analysis and gene ontology annotation

In order to explore the differences in biological processes among different FASM patterns, Gene set variation analysis (GSVA) was carried out using the GSVA package [34]. Moreover, The Kyoto Encyclopedia of Genes and Genomes (KEGG) pathways related to different patterns were identified using clusterProfiler R package with a false discovery rate (FDR) cutoff below 0.01 [35].

### mRNA stemness index (mRNAsi) computation

The transcriptional mRNAsi index for each LIHC sample was computed using One-class logistic regression (OCLR), which is based on pluripotent stem cell samples and strongly correlated with stem cell features. This index can be applied for cancer stemness predictions [36]. Both prognostic value of mRNAsi as well as the Spearman correlation between FASM subtypes and mRNAsi indices across all 363 patients with LIHC were analyzed.

### PPI network analysis

STRING database (https://cn.string-db.org/), a functional protein-related network, assembles all known and predicted proteins [37]. The PPI network interactions file with a medium confidence score (> 0.4) was available. Furthermore, cytoscape software (version 3.8.2), a common open source, was used to beautify and analysis interaction network [38]. To explore hub genes, the cytoHubba-MCC was used [39].

### Single cell RNA-seq data integration and analysis

Normalized data were integrated using the "Findinintegrationanchors" function in the Seurat software package [40]. Then, the data were reduced dimensionality and performed PCA. “FindNeighbors” and “FindCluster” were performed to analysis LIHC cell clusters. The tSNE was utilized to visualize the cell clusters. The cell cluster markers were acquired by screening the literature and retrieving the CellMarker 2.0 database (http://bio-bigdata.hrbmu.edu.cn/CellMarker/) to annotate cell type [41]. Based on scRNA-seq data, the R package CellChat can infer, visualize, and analyze of intercellular communication and describe the interactions among ligands, receptors and secreted factors [42]. To investigate the possible communication between CSCs and other cells, the ligand-receptor and secretion interactions between cell types were analyzed by using Cellchat.

### Cell culture and functional assays

L02, HepG2, HCCLM3 cell lines were supplied by American Type Culture Collection or Cell Bank, Shanghai Institutes for Biological Sciences, Chinese Academy of Sciences. These cells were authenticated using Short Tandem Repeat (STR) analysis. L02 cells were cultured in RPMI 1640 medium (10% FBS and 1% streptomycin/penicillin) at 37 °C under 5% CO_2_. HepG2, HCCLM3 cells were cultured in DMEM media at same growth conditions. The colony formation ability of HCC cell lines was detected by plate cloning experiment. 500 cells were colonized into 6-well plates with 20% FBS medium. After culturing for 7 days under the condition of 37 °C and 5% CO_2_, colonies were terminated in 4% paraformaldehyde (PFA) and dyed with crystal violet for 2 min. Then the colonies were captured by microscopy. The cell counting kit-8 (CCK-8) was utilized to detect cell proliferation. 10000 cells were inoculated in 96-well plates with condition medium 48 h. The medium absorbance was examined using microplate reader. HCC cells were plated into 6-well plates with low adhesion (500 cells per well). Then we added 2 ml DMEM/F-12 medium containing 1% B-27, 1% N-2, 20 ng/mL epidermal growth factor, and 10 ng/ml basic fibroblast growth factor into 6-well plates. Replace the medium every 3 days. The stem cell spheres were captured by microscopy.

### Immunohistochemical staining

Tumor tissues from 5 patients with HCC in Chongqing University Cancer Hospital were collected. And the clinical characteristics of 5 samples in Supplementary Materials (Additional file 5: Table S4). 4% PFA was used to fix HCC tissue for 24 h, the paraffin embedding was processed according to standard procedures. The embedded tissues were cut to a thickness of 8 μm and placed on a slide. After deparaffinizing, tissues were performed antigen repair in citrate buffer in microwave for 15 min at least. Following manufacturer’s instructions, the treated sections with anti-ACACA antibody were incubated overnight at 4 °C, washed thrice with phosphate-buffered saline (PBS) for 5 min each, and reacted with the appropriate concentration of secondary antibodies for 1 h at 25 ℃. Immunohistochemical microscopic images were obtained utilizing optical microscope.

### Immunofluorescence

To prepare the cells for imaging, cell and climbing placed in 24-well plates and washed twice with PBS before being fixed in 4% PFA for a duration of 15 min. Subsequently, the climbing with cells underwent three washed with PBS and were permeabilized using a solution containing 0.3% Triton-X100/PBS at 25 ℃ for 15 min, followed by another round of triple washing with PBS. For blocking purposes, the cells were treated with a solution consisting of 5% bovine serum albumin (BSA) at 25 ℃ for 1 h after which they were incubated overnight at 4 °C in a mixture containing anti-ACACA antibody. Following washed with PBS to remove any excess primary antibody, secondary antibody was applied to the cells and allowed to incubate for an hour at room temperature. After another round of triple washing with PBS, phalloidin-iFluor staining was carried out on the cells for half an hour and DAPI staining at room temperature for ten minutes.

### Quantitative reverse transcription polymerase chain reaction (qRT-PCR)

The total RNA from cells was extracted using TRIzol. The transcriptional level of ACACA was detected via qRT-PCR with following program: 95 ℃ for 30 s, 40 cycles of 95 °C for 5 s, and 60 °C for 30 s. The reaction system consisted of 5 μL SYBR Green (Foregene, Chengdu, China), 1 μL each primer, 1 μL cDNA, and 2 μL diethylpyrocarbonate (DEPC) water.

ACACA forward primer: 5′ - AGGAGCTGTCTATTCGGGGT - 3′,

reverse primer:5′ - ATGTCTGGCTTGCACCTAGTA - 3′;

and GAPDH forward primer: 5′ - GGTATGACAACGAATTTGGC - 3′,

reverse primer: 5′ - GAGCACAGGGTACTTTATTG - 3′.

### Statistical analyses

Unpaired student’s t test was applied to analysis two sets of experiments, one-way ANOVA was utilized for 3 or more groups. Data was analyzed using GraphPad Prism software 8.0 or R version 4.3.0. Each experiment was repeated three times, unless otherwise specified in the figure legends.

## Results

### Identification and characterization of FASM genes in HCC progression

**Fig. 1:**
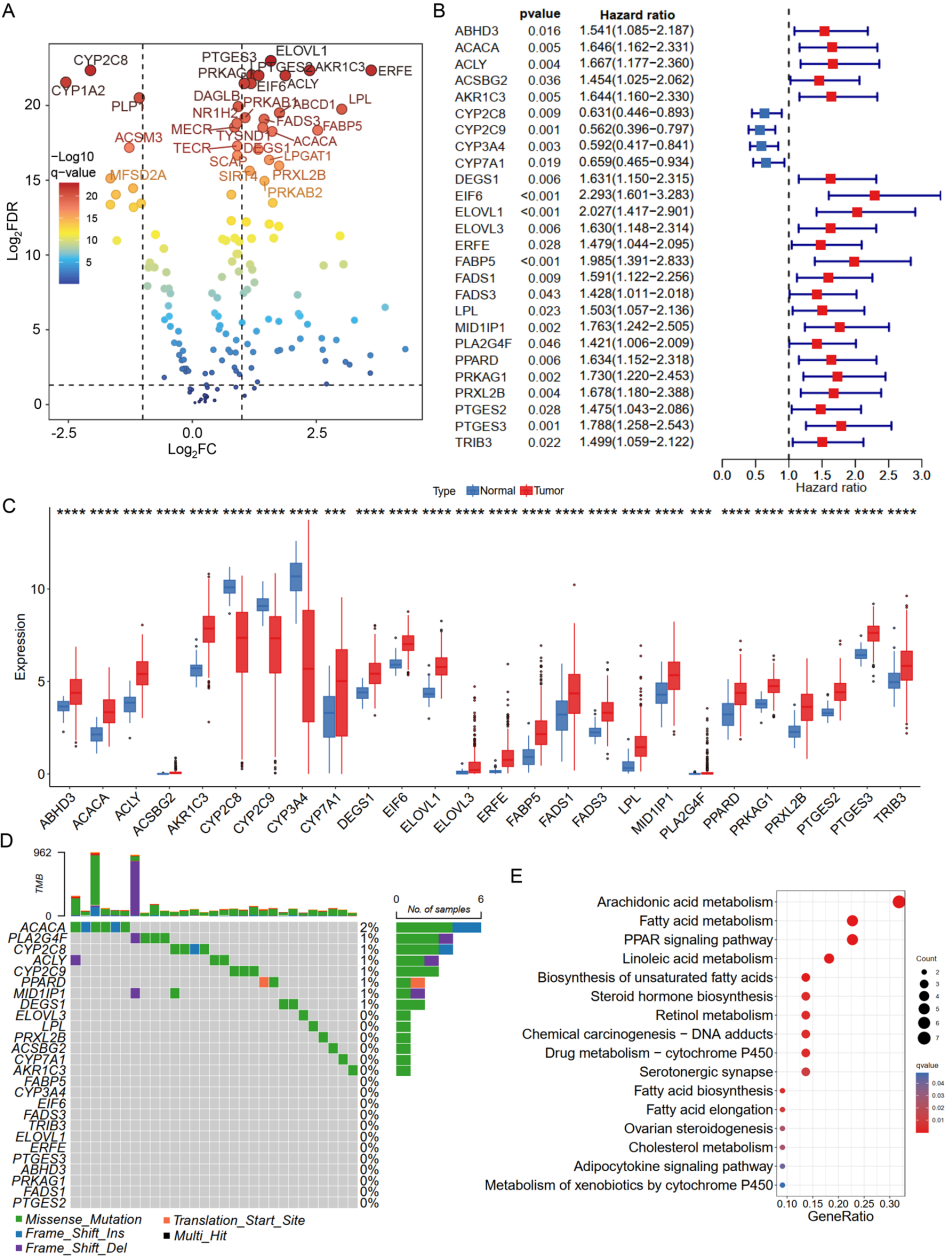
Characterization of FASM-related genes in HCC. **A** Volcano map of FASM genes in the HCC patients and healthy controls. **B** Univariate Cox regression analysis of 26 FASM genes related to clinical prognosis in HCC. **C** The 26 FASM genes present differential expression between HCC and normal tissues. **D** Waterfall plot shows the mutational landscape of the 26 FASM genes and clinicopathological characteristics. **E** KEGG pathway enrichment analysis of 26 FASM genes. *p < 0.05; **p < 0.01; ***p < 0.001; ****p < 0.0001; *ns* no statistical significance

The summary clinical characteristics of 664 HCC samples was presented in Table 1. Comprehensive analysis of FASM genes was performed based on multi-group data of TCGA-LIHC cohort (Additional file 4: Table S3). Differential gene analysis from transcriptome data revealed that 73 of 203 FASM genes were upregulated or downregulated in HCC (Fig. 1A; Additional file 1: Fig S1A). Univariate Cox regression results suggested that 26 out of 73 FASM differential expression genes were associated with prognosis in HCC (Fig. 1B). The expression of 26 FASM genes displayed particularly significant heterogeneity between normal and HCC tissues (Fig. 1C). The mutational landscape for the 26 FASM genes was displayed in a waterfall plot. Eight out of the 26 FASM genes owned a high mutation frequency and were closely relevant to progression or recrudesce in HCC (Fig. 1D). Moreover, the kyoto encyclopedia of genes and genomes (KEGG) result showed that 26 FASM genes were concentrated in the arachidonic acid metabolism, fatty acid metabolism and PPAR signaling pathway (Fig. 1E). Thus, these results suggest that the changes of FASM genes expression level may regulate the development and progression of HCC.

**Table 1:** The summary clinical characteristics of 664 HCC samples

Characteristic	All patients (case%)
Total	664
Gender	
Male	460 (69.27%)
Female	204 (30.72%)
Age	
≤ 50 years	106 (15.96%)
> 50 years	558 (84.04%)
Race	
Asian	406 (61.14%)
White	219 (32.98%)
Others	39 (5.87%)
Treatment history	
Yes	44 (6.63%)
No	349 (52.56%)
Not reported	271 (40.81%)
Stage	
Stage I	227 (34.19%)
Stage II	207 (31.17%)
Stage III	171 (25.75%)
Stage IV	27 (4.07%)
Not reported	32 (4.82%)

### FASM patterns in HCC

**Fig. 2:**
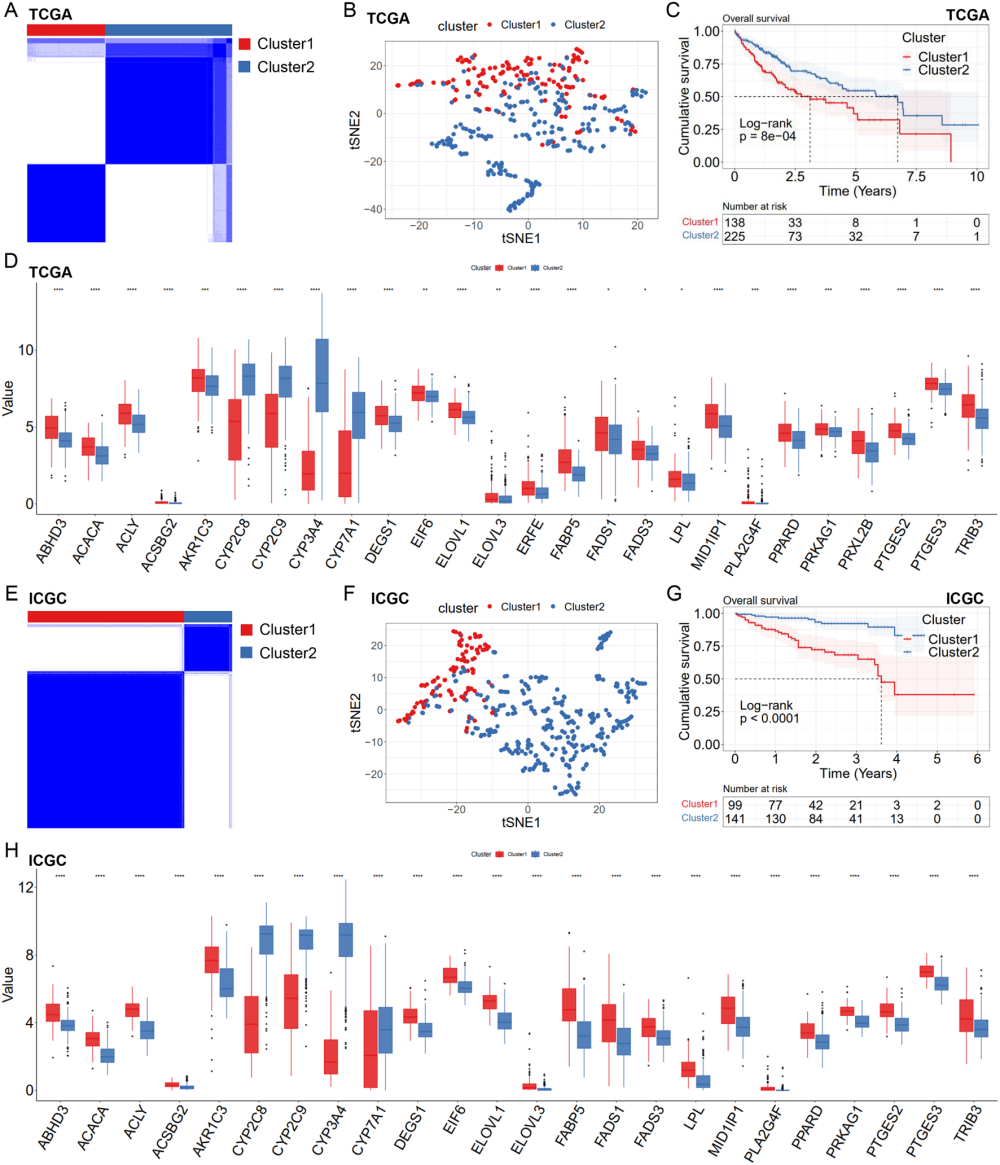
Patterns of FASM and their prognostic value in HCC. **A** Two FASM patterns were determined by unsupervised clustering in HCC from TCGA. **B** tSNE verified the two patterns in TCGA-LIHC. **C** Survival analysis showed that there were observably different survival outcomes in TCGA-LIHC between the patients based on two clusters. **D** Box plot of differential expression of 26 FASM genes in two clusters from TCGA-LIHC. **E** Two patterns of FASM were identified by unsupervised clustering in ICGC-LIRI. **F** tSNE confirmed the two patterns in ICGC-LIRI. **G** Survival analysis showed that there were observably different survival outcomes in ICGC-LIRI between the patients based on two clusters. **H** Box diagram of differential expression of 26 FASM genes in two clusters from ICGC-LIRI. *p < 0.05; **p < 0.01; ***p < 0.001; ****p < 0.0001; *ns* no statistical significance

According to the RNA-seq data of 26 FASM genes and clinical data from TCGA-LIHC, unsupervised clustering was performed by using ConsensusClusterPlus package to classify LIHC patients into two different clusters (Clusters1 and Clusters2) (Fig. 2A). The differences between the two FASM models were firstly evaluated by PCA algorithm and tSNE algorithm. It was observed that there were obvious differences in transcriptional profile among the FASM clusters (Fig. 2B; Additional file 1: Fig S2A). We then assessed the clinical prognostic value of FASM patterns in HCC patients via a survival analysis. Patients corresponding to two clusters exhibited a significant variation in survival from TCGA dataset (Fig. 2C). Based on the data of TCGA-LIHC, the expression of 26 FASM genes was prominently different between the two clusters (Fig. 2D; Additional file 1: Fig. S2B). To explore the stability and applicability of two FASM patterns in HCC, the unsupervised clustering analysis was repeated by using LIRI-JP cohort from ICGC. The result showed that populations could be well divided into two categories (Fig. 2E). The PCA and tSNE analysis results confirmed the two disparate patterns of FASM in HCC (Fig. 2F; Additional file 1: Fig. S2C). And there was also an obvious difference in survival from ICGC dataset between the patients of two clusters (Fig. 2G). Based on ICGC-LIRI expression profiling data, 26 FASM genes in the two clusters were greatly differentially expressed (Fig. 2H; Additional file 1: Fig. S2D).

### Correlation of the FASM patterns with immune cell infiltration

**Fig. 3:**
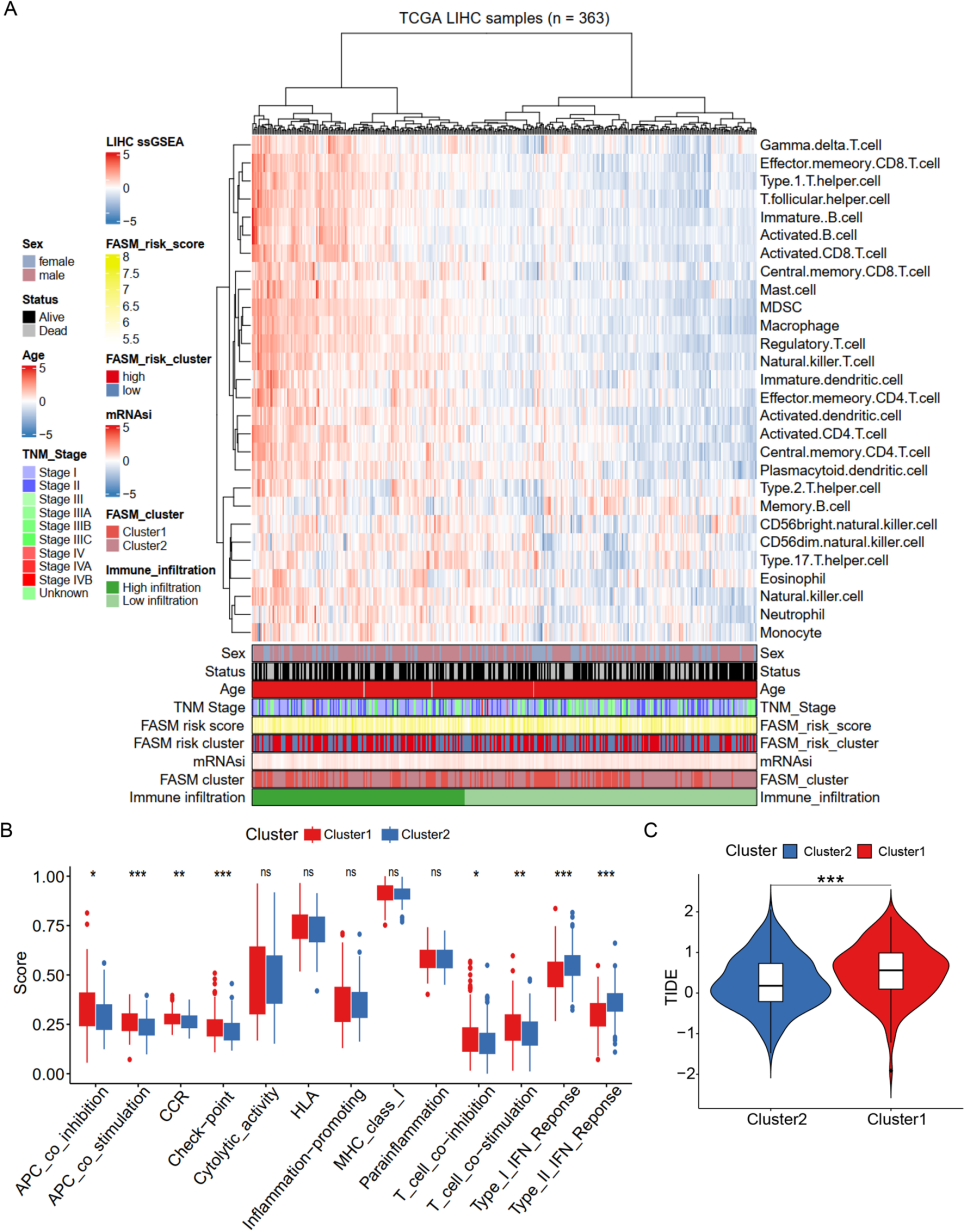
Correlation between FASM patterns and immune infiltrating cells. **A** Heatmap represents the relationship between TIME infiltration and clinical pathological parameters as well as FASM riskScore, and subtypes. **B** Differences of the immunologic function in two FASM subtypes. **C** Difference analysis of TIDE scores in two FASM subtypes. *p < 0.05; **p < 0.01; ***p < 0.001; ****p < 0.0001; *ns* no statistical significance

To explore the immune characteristics in FASM patterns, we estimated the immune infiltration characteristics of 28 immune cell proportions in FASM patterns and made visual analysis via heatmap (Fig. 3A). In comparison, some kinds of tumor immunosuppression cells were more abundant in Cluster1, including regulatory T cells and macrophages, indicating that the patients of Cluster1 subtype presented an immunosuppressive microenvironment that promoted tumor progression. We then explored enrichment fraction of the immune cells and their immune pathway activity. These two models showed entirely different immune properties. As in TCGA, regulatory T cells and M0 macrophages were mainly concentrated in Cluster1, while activated NK cells and proinflammatory M1 macrophages mostly concentrated in Cluster2 (Additional file 1: Fig. S3A). Consistently, similar infiltrative features of regulatory T cells and M0 macrophages in Cluster1 were also observed in ICGC (Additional file 1: Fig. S3B). In immune pathway activity, Cluster1 possessed higher CCR, Check-point and T cell co-inhibition activity. Whereas, Type I and II IFN Reponses were mainly enriched in Cluster2 (Fig. 3B). At last, we evaluated the sensitivity of FASM patterns to immunotherapy. The results displayed that Cluster1 had enhanced TIDE, indicating that Cluster1 had a higher probability of immune escape and a poor response to immunotherapy (Fig. 3C). Besides, the biological functions of FASM Pattern were further investigated. We assessed the enrichment scores of the Hallmark pathways and KEGG pathways in the FASM cluster subtypes by GSVA. The two subtypes exhibited different pathways, including pathogenic escherichia coli infection, lysine degradation and adipocytokine signaling pathway (Additional file 1: Fig. S3C).

### Correlation of the FASM patterns with mRNAsi

**Fig. 4:**
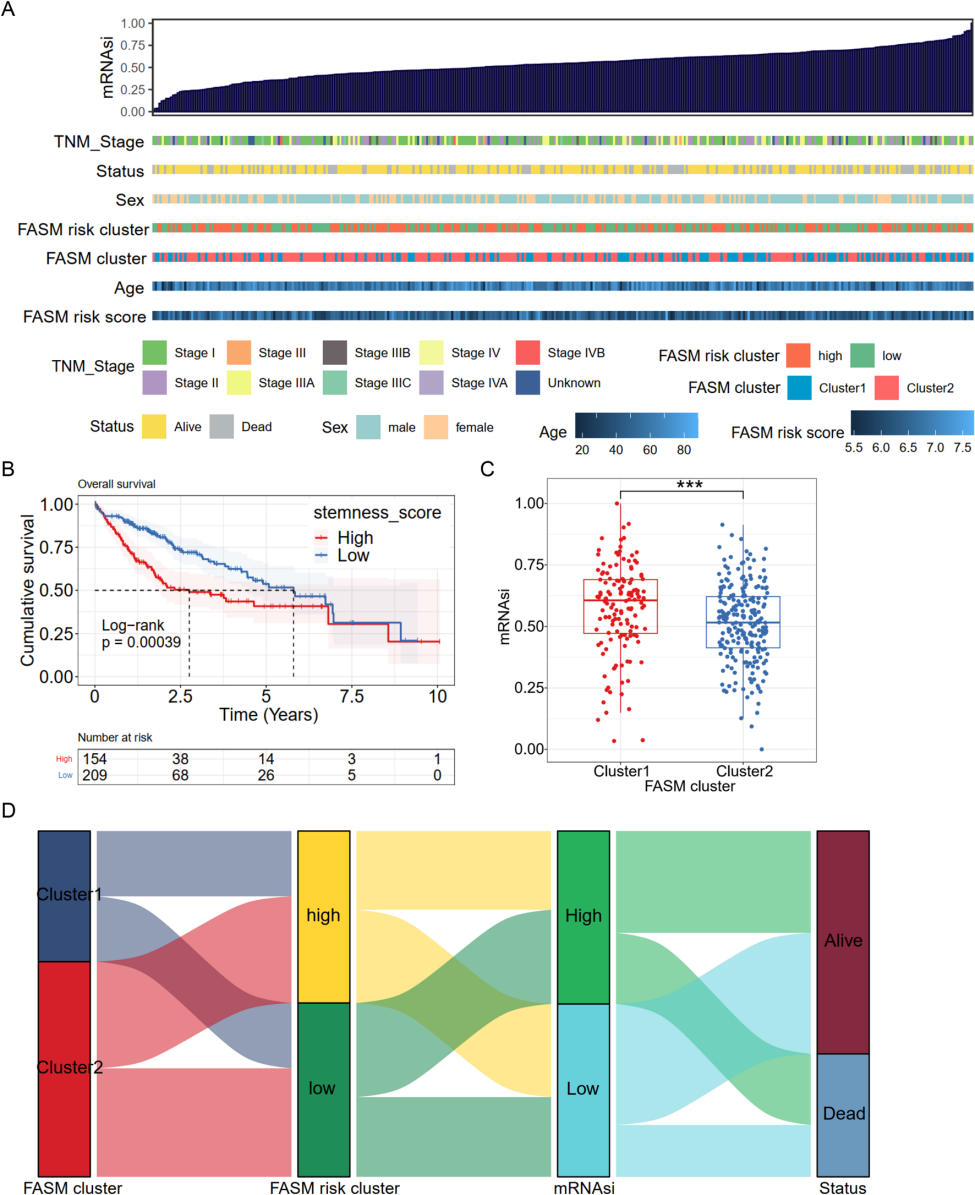
Correlation between FASM patterns and mRNAsi. **A** The overview of the relevance of mRNAsi to clinical features, FASM subtypes and risk groups. **B** Kaplan–Meier survival analysis displayed that high mRNAsi is associated with a poor prognosis in HCC patients. **C** Box plot of mRNAsi comparison between two FASM subtypes. **D** The relationships between subtypes, risk, mRNAsi and status of patients. *p < 0.05; **p < 0.01; ***p < 0.001; ****p < 0.0001; *ns* no statistical significance

Fatty acid synthase expression occurs predominantly in proliferating fetal cells [43], implying that reactivation of FA synthesis in cancer cells may be a signal to return a stemness maintenance status. By the OCLR algorithm, mRNAsi of each LIHC patients was estimated on basis of the gene expression profiles, and then the correlations between mRNAsi and the FASM subtypes was detected (Fig. 4A). In addition, Kaplan–Meier analysis showed that LIHC patients with high-mRNAsi experienced shorter OS than low-mRNAsi patients (Fig. 4B). By sequencing mRNAsi from low (left) to high (right), we found that FASM Cluster1 was largely enriched in high mRNAsi regions, while FASM Cluster2 presented the lowest mRNAsi, which was demonstrated by the comparative analysis (Fig. 4C), and the distribution of these patients in FASM patterns were displayed in Sankey diagram (Fig. 4D).

### Chemotherapy sensitivity among FASM patterns

**Fig. 5:**
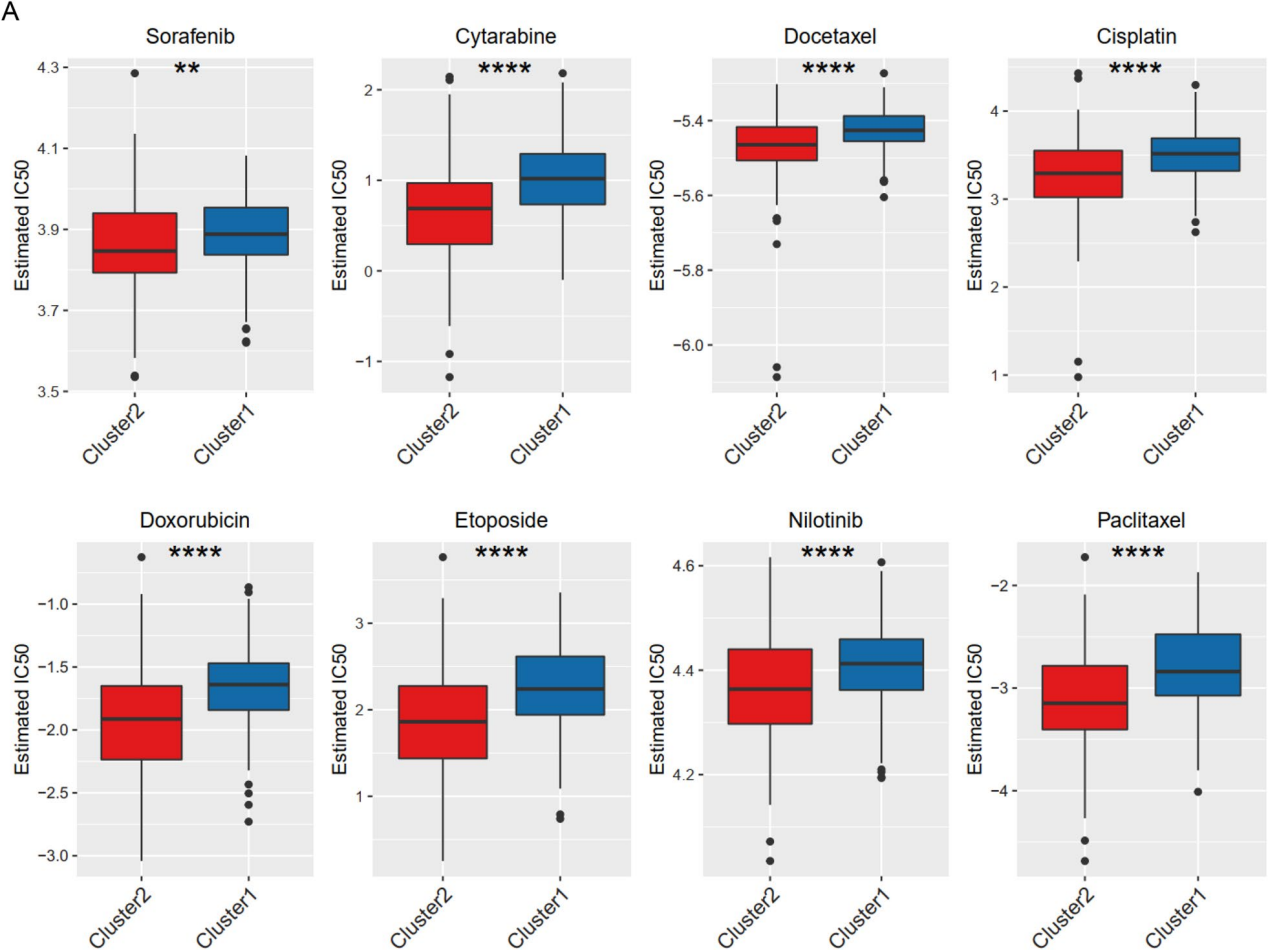
Response to chemotherapeutics for FASM subtypes in HCC. **A** Response to 8 common chemotherapeutics for Cluster1 and Cluster2 in HCC. *p < 0.05; **p < 0.01; ***p < 0.001; ****p < 0.0001; *ns* no statistical significance

At present, surgery and systemic chemotherapy are the main clinical treatment options for HCC patients. Therefore, we calculated half maximal inhibitory concentration (IC50) values for chemotherapy sensitivity evaluations of several chemotherapeutics drugs by pRRophetic algorithm and compared IC50 values among FASM patterns. The results showed that estimated IC50 values of Sorafenib, Cytarabine, Docetaxel, Cisplatin, Doxorubicin, Etoposide, Nilotinib and Paclitaxel were substantially lower in Cluster2, suggesting that Cluster2 might be more sensitive to these drugs. On the contrary, Cluster1 were more tolerant to these drugs (Fig. 5A). The study of chemotherapy sensitivity among FASM patterns provided a certain theoretical basis for clinical medication.

### FASM risk prognostic model and clinical nomogram construction

**Fig. 6:**
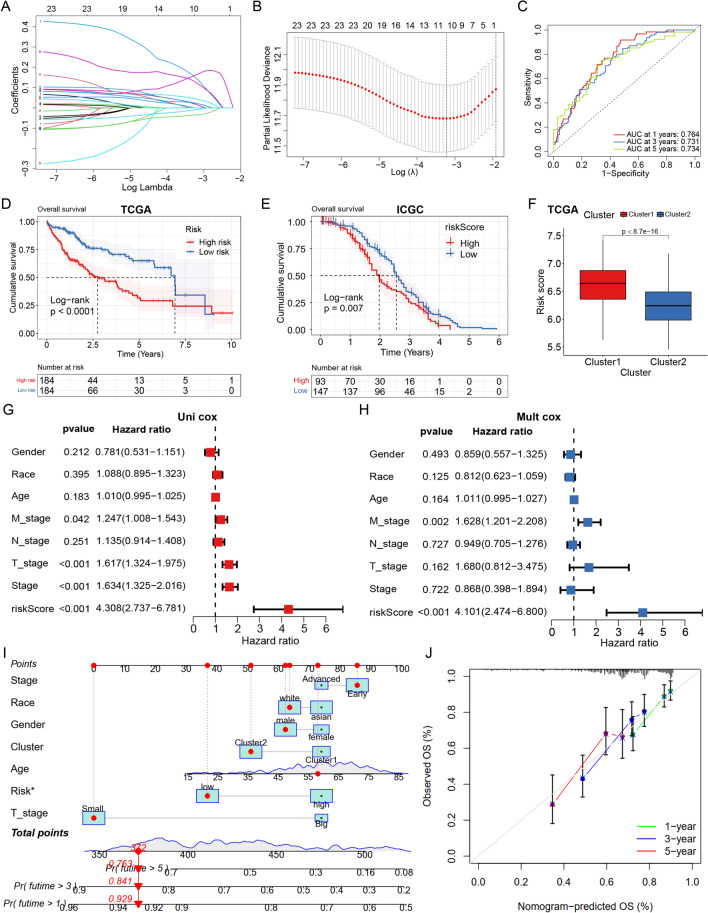
Construction risk model of FASM genes and validation of its prognostic value. **A** LASSO analysis. **B** Screening of the optimal parameter at the vertical lines. **C** The ROC values internally validated by the proposed model. **D**, **E** Kaplan–Meier survival curves of FASM risk in TCGA. **F** Correlation of risk and subtypes in TCGA. **G**, **H** Univariate Cox and Multivariate Cox analysis of common clinicopathological parameters and FASM risk. **I** Nomogram combining common clinical parameters and FASM. **J** Calibration curves for nomograms

Based on 26 FASM genes, Lasso analysis and multivariable Cox regression analysis were performed. As a result, 11 candidates (EIF6, ELOVL3, LPL, CYP2C9, PRXL2B, ELOVL1, ACACA, CYP7A1, DEGS1, PTGES3, AKR1C3) were marked as independent prognostic genes (Fig. 6A, B). The ROC curves displayed that the AUC of the risk cohort was > 0.7 at 1, 3, 5 years (Fig. 6C; Additional file 1: Fig. S4A). LIHC patients were divided into low- or high-risk groups, and the prognosis of patients in the high-risk group was poor. (Fig. 6D, E). Importantly, Cluster1 exhibited a higher risk score compared to Cluster2 from TCGA and ICGC (Fig. 6F; Additional file 1: Fig. S4B), indicating that FASM subtypes had a good consistency among the risk prognostic model of FASM. To establish a more practical and reliable nomogram, some familiar clinicopathological indicators were introduced (gender, race, age and tumor stage). Univariate Cox and Multivariate Cox analysis revealed that FASM riskScore was a risk factor for the prognosis of HCC patients (Fig. 6G, H). In view of the high correlation between FASM riskScore and prognosis of HCC patients, we integrated conventional clinicopathological parameters with FASM riskScore to establish a new nomogram (Fig. 6I). We first used the ROC curve to assess the AUC values of various indicators to predict the prognosis of HCC patients, and the results showed that the FASM riskScore was greatly better than other clinicopathologic characteristics. And this nomogram further improved the accuracy of predicting the prognosis of HCC patients (Additional file 1: Fig. S4C). Furthermore, the calibration curves further illustrated the good accuracy and robustness of nomogram in evaluating patient survival at 1, 3, and 5 years (Fig. 6J). Interestingly, the FASM high-risk group exhibited the same characteristics of immune infiltration and drug tolerance as Cluster1 (Additional file 1: Fig. S5A–C; Additional file 1: Fig. S6A). Those data indicated that 11 prognostic signatures could serve as reliable predictors of OS in LIHC patients.

### Hub genes correlation analysis

**Fig. 7:**
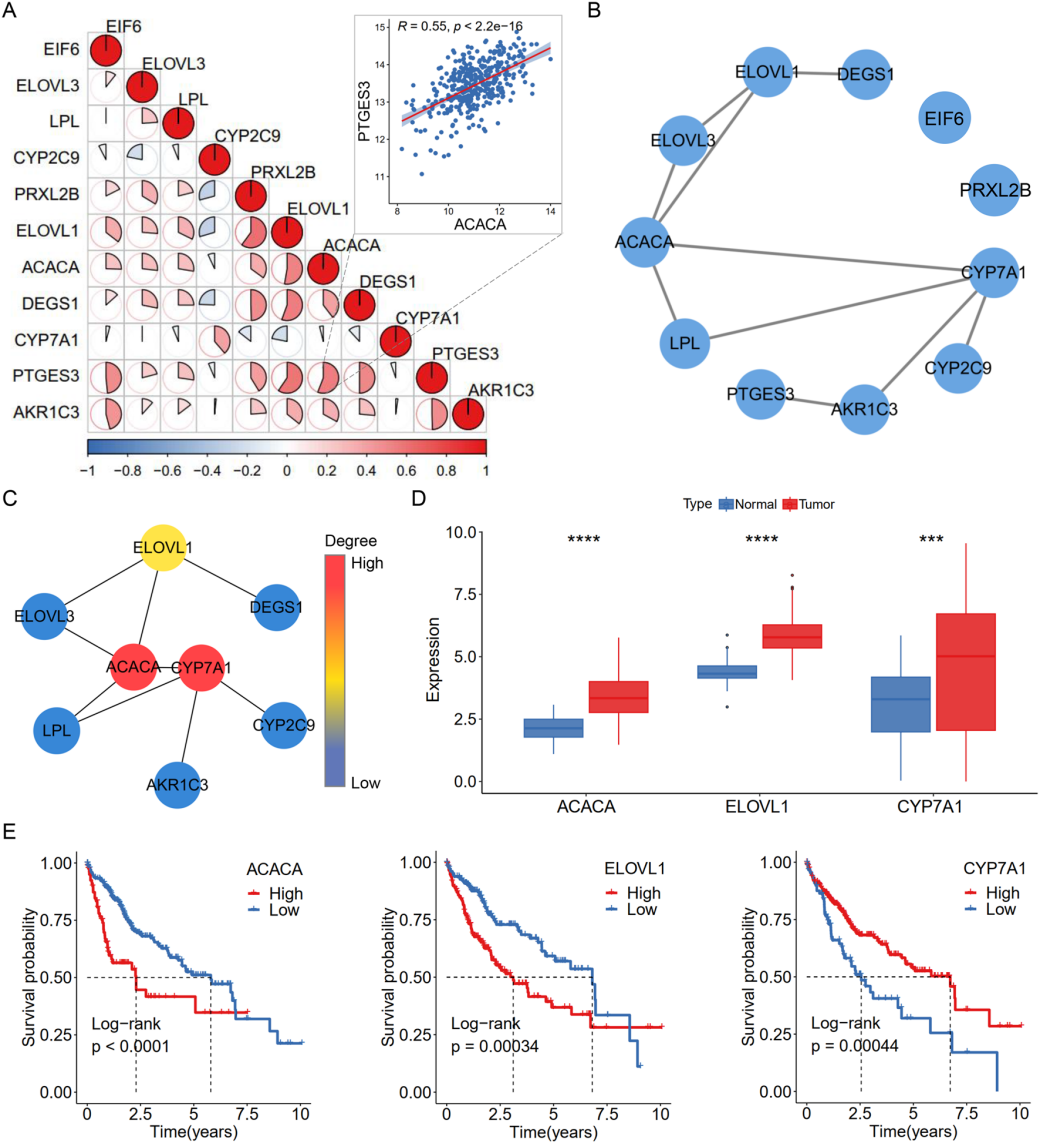
Identification of hub genes in FASM. **A** Correlation analysis of the 11 FASM risk genes. **B** PPI network of the 11 FASM risk genes. **C** Identification of hub genes in the network via Cytohubba-MCC. **D** Expression of ACACA, ELOVL1 and CYP7A1 in HCC and normal tissues. **E** Kaplan–Meier survival analysis displayed high the expression of ACACA, ELOVL1 and CYP7A1correlated with poor prognosis in HCC patients. *p < 0.05; **p < 0.01; ***p < 0.001; ****p < 0.0001; *ns* no statistical significance

To identify the key regulatory genes of FASM, we evaluated the correlations among FASM risk genes and found obvious synergistic effects (Fig. 7A). Whereafter, a PPI network was established using the STRING database, and 3 genes with a degree > 2 by the cytoHubba-MCC plugin were identified (Fig. 7B, C). The expression of CYP7A1, ELOVL1 and ACACA were higher in LIHC tumor tissues than normal tissues (Fig. 7D). In survival analysis, ACACA with high expression group had the worst prognosis (Fig. 7E). Those findings indicated that ACACA was the key regulatory gene of FASM in HCC for further study.

### The biological characteristics of ACACA in LIHC

**Fig. 8:**
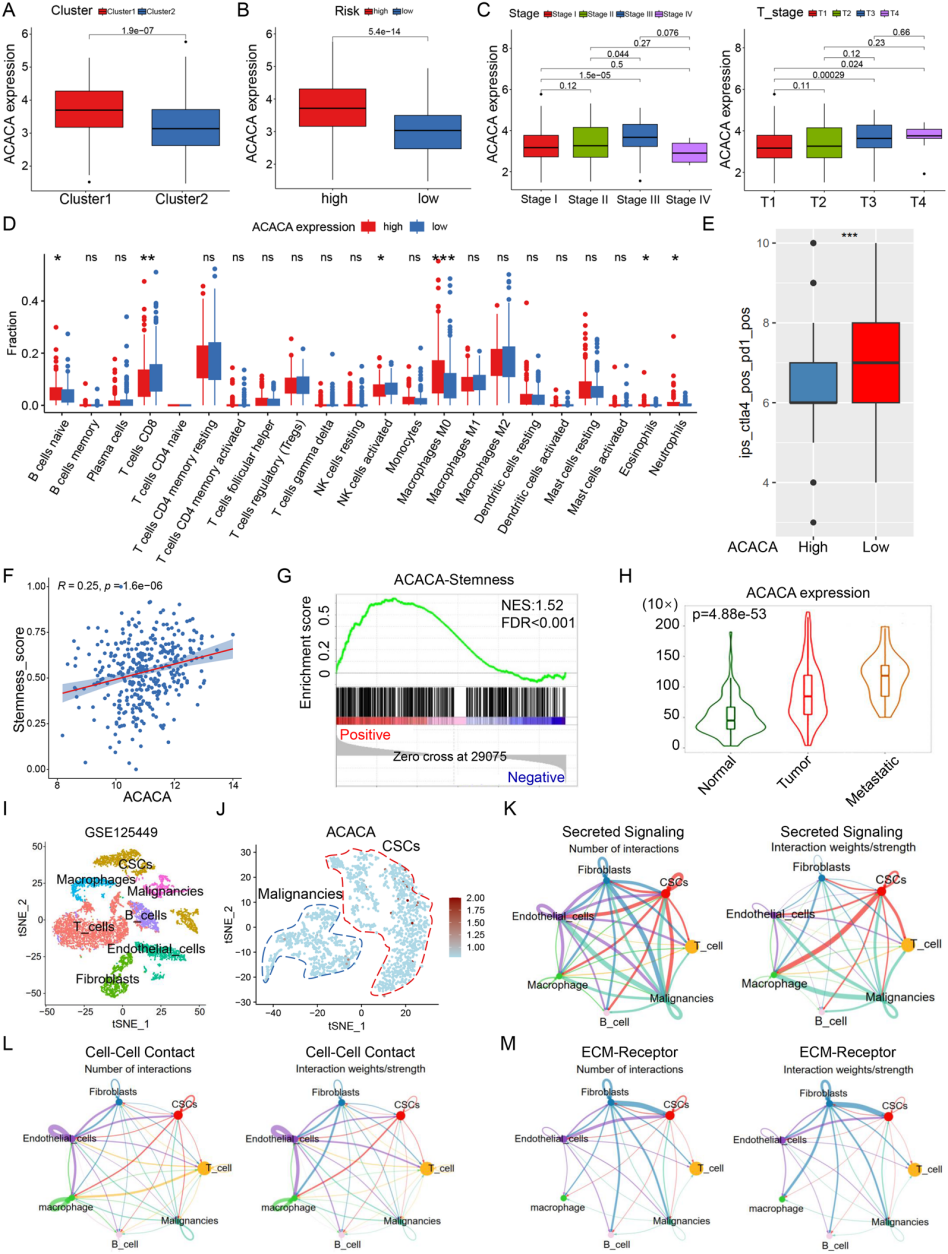
Correlation of ACACA expression with clinical features, immune response, stemness, metastasis, and cellular interactions in HCC. **A**, **B** Expression of ACACA in FASM patterns. **C** Expression of ACACA in stages of HCC. **D**, **E** Correlation analysis between the ACACA expression and immune cell infiltration as well as immune response. **F**, **G** Correlation analysis between the ACACA expression and HCC stemness. **H** Expression of ACACA in metastasis of HCC. **I** Cell subtypes were identified by marker genes in scRNA sequencing. **J** Expression of ACACA in scRNA sequencing. **K** Cellchat analysis of interactions between cell subsets via Secreted Signaling. **L** Cellchat analysis of interactions between cell subsets via Cell–Cell Contact. **M** Cellchat analysis of interactions between cell subsets via ECM-Receptor. *p < 0.05; **p < 0.01; ***p < 0.001; ****p < 0.0001; *ns* no statistical significance

We further investigated the pathological mechanisms of ACACA in HCC progression. The differential expression of ACACA in FASM risk and cluster subtypes were calculated. The expression of ACACA in Cluster1 and FASM high-risk group was notably higher than Cluster2 and FASM low-risk group (Fig. 8A, B). ACACA also showed differential expression in different stages of tumors, especially in stage III and T3 stage (Fig. 8C). ACACA had the most significant positive correlation with M0 macrophages. Meanwhile, ACACA was negatively correlated with CD8 T cells (Fig. 8D). Similarly, LIHC patients with high ACACA expression had lower responsiveness to CTL4 and PD1 (Fig. 8E). Likewi se, ACACA was significantly positively correlated with HCC stemness and metastasis (Fig. 8F, H). Finally, we detected the expression of ACACA in scRNA sequencing of patient with HCC from GSE125449. By removing low-quality cells, normalizat ion, integration, and PCA, we divided the cells into 10 clusters (Additional file 1: Fig. S7A), which were then annotated for analysis. Finally, 7 cell types, including T cells (clusters 0, 8), Cancer stem cells (CSCs) (clusters 1, 7), Fibroblasts (cluster 2), Endothelial cells (cluster 3, 9), Macrophages (cluster 4), B cells (clusters 5) and Malignancies (clusters 6) (Fig. 8I; Additional file 1: Fig. S7B) were identified. The results indicated that the expression level of ACACA in CSCs was much higher than that in malignant cells (Fig. 8J).

The communication properties of CSCs with highly expressing ACACA and other cell types were also investigated. We used Cellchat to test Secreted Signaling, Cell–Cell Contact and ECM-Receptor interactions between different cells (Fig. 8K–M). The results suggested that Secreted Signaling-mediated cellular interactions mainly existed in the MIF signaling pathway (MIF-CD74 + CXCR4) (Additional file 1: Fig. S7C–D). APP-CD74 pathway contributed the most to Cell–Cell Contact (Additional file 1: Fig. S7E–F). In addition, VTN-(ITGAV-ITGB1) pathway was dominant in ECM-Receptor (Additional file 1: Fig. S7G, H). The above results demonstrated that ACACA may motivate the stemness of HCC through the fatty acid pathway, thereby leading to immune escape and tumor metastasis through CD74 and ITGB1 signaling.

To gain a more comprehensive understanding of ACACA's role in cancer, the TCGA and Genotype-Tissue Expression (GTEx) databases were used to estimate the expression level of ACACA in pan-cancer compared with normal tissues. ACACA is highly expressed in most cancers, including CESC, CHOL, COAD, ESCA, LIHC, PRAD, READ, STAD, UCEC. On the contrary, low ACACA expression was found in GBM, KICH, KIRC and THYM (Additional file 1: Fig. S8A). To further understand the effect of ACACA on patient prognosis, univariate Cox regression was employed to analyze the prognosis across 33 cancer types. The forest plot revealed that the downregulation of the ACACA expression was significantly related to the prolongation of OS time in ACC (HR = 2.79[95% CI 1.26–6.21], p = 0.012), BLCA (HR = 1.36[95% CI 1.01–1.82], p = 0.041), KICH (HR = 9.63[95% CI 1.2–77.57], p = 0.033), LIHC(HR = 1.65[95% CI 1.16–2.33], p = 0.005), MESO (HR = 1.84[95% CI 1.14–2.97], p = 0.012) and UVM (HR = 2.92[95% CI 1.14–7.43], p = 0.025) (Additional file 1: Fig. S8B). Then genomic variant analysis of ACACA showed that ACACA variants are ubiquitous in pan-cancer. Meanwhile, the maximum frequency of ACACA mutation (> 10%) were found in SKCM and UCEC patients, but the frequency of ACACA changes in KICH and MESO patients were minimum (Additional file 1: Fig. S8C).

Next, the types and loci of ACACA alteration were also validated (Additional file 1: Fig. S8D). To further comprehend the role of ACACA mutation in pan-cancer, we assessed the association of ACACA expression with tumor mutation burden (TMB) and microsatellite instability (MSI). To be expected, ACACA expression was positively related to TMB in most cancers, including LIHC, but it showed a negative correlation with BRCA and ESCA (Additional file 1: Fig. S8E). When we compared the relationship of ACACA positive expression and MSI in most cancers, the noted result was positively correlated with GBM, KIRC, LIHC, LUAD, LUSC, STAD, UCEC and CESC, and negatively correlated with BRCA, DLBC, HNSC and THCA (Additional file 1: Fig. S8F). All the above, these results suggested that ACACA was associated with tumorigenesis and tumor immunity.

### Multilevel expression verification and functional exploration of ACACA in vivo and in vitro

**Fig. 9:**
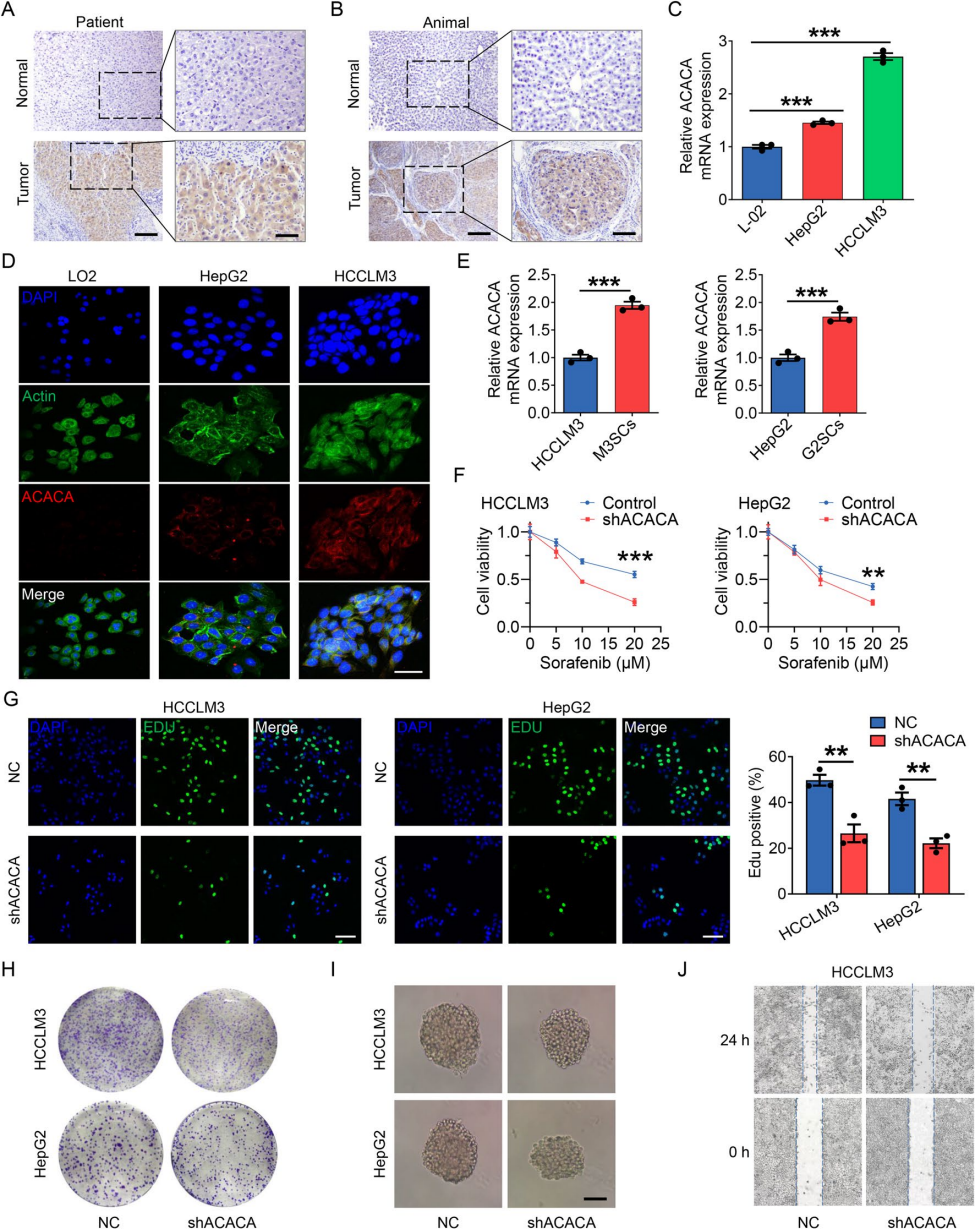
Multiple validation that ACACA promotes tumor progression in vivo and in vitro*.* **A**, **B** Expression of ACACA in HCC patient samples and SD rat orthotopic liver cancer model. Bar, 100 µm. Zoom bar, 50 µm. **C**, **D** Expression of ACACA in HCC cell lines. Bar, 25 µm. **E** Expression of ACACA in HCC stem cell and their parental cell. **F** CCK8 detection: the effect of ACACA knockout on the sensitivity of sorafenib. **G**, **H** Edu and colony forming assay: the effect of ACACA knockdown on the proliferation of HCC cell lines. Bar, 50 µm. **I** Sphere enrichment assay: the effect of ACACA knockdown on the stemness traits of HCC cell lines. Bar, 50 µm. **J** Wound healing assay: the effect o of ACACA knockdown on the migration capability of HCC cell lines. *p < 0.05; **p < 0.01; ***p < 0.001; ****p < 0.0001; *ns* no statistical significance. n = 3 per group

To verify the important role of ACACA in HCC, we used the HCC patient samples and SD rat orthotopic liver cancer models. Immunohistochemical staining displayed that ACACA expression was higher in HCC compared to normal tissues (Fig. 9A, B). Analogously, qRT-PCR assay and immunofluorescence staining revealed the expression level of ACACA was obviously higher in HCC cell lines (HepG2 and HCCLM3) than in liver cell (L02) (Fig. 9C, D). Particularly, the expression of ACACA was significantly up-regulated in induced liver cancer stem cells (M3CSs and G2CSs) compared to their parent cells (Fig. 9E). To better understand the function of ACACA in vitro, we analyzed the oncogenic phenotype of HCCLM3 and HepG2 cells with ACACA knockdown (shACACA). CCK8, Edu and colony formation assays manifested that the depletion of ACACA impaired HCC cell proliferation (Fig. 9F–H). Sphere formation assay and wound healing were carried out to evaluate the stemness traits and metastatic potential of ACACA. The results suggested that the sphere sizes and migration distance were noticeably shortened after shACACA transfections in HCC cells, exhibiting a suppression of stemness properties and metastatic potential in HCC cells upon ACACA depletion (Fig. 9I, J).

## Discussion

Hepatocellular carcinoma (HCC), as the most common type of liver malignancy, possesses a high possibility of metastasis and recurrence. Unfortunately, most patients with HCC are diagnosed at later stages, which will miss the opportunity for a good cure and result in a poor prognosis [44]. Accumulating evidence suggests that the aberrant activity of FASM metabolic pathways is critical to tumor cell fate and progression in HCC. However, the metabolic characteristics and role of FASM-related pathways in HCC remain largely undefined. Here, we identified the molecular characteristics, physiologic function and prognosis value of twenty-six FASM-related differential genes in HCC. Specially, by the in vivo and in vitro experiments, we proved that ACACA, a rate-limiting enzyme of FA metabolism, govern cancer stemness and immune escape and promote cancer progression.

Recent studies have shown that systematic analyses of specific genomes have yielded satisfactory results in predicting cancer prognosis [45, 46]. Despite this, reliable molecular signature analysis and HCC prognostic models based on FAs synthesis and metabolism gene sets are still lacking. In this study, using unsupervised clustering analysis, we classified HCC patients into two groups based on clinical data and expression data from TCGA. Based on the data from LIRI-JP in the ICGC, we verified our findings in an Asian population. We found significant expression differences of twenty-six FASM genes between the two subtypes. Patients in Cluster1 had a stronger capacity in lipid mobilization and a worse prognosis. Consist with other studies, elevated level of lipid mobilization protected tumor cells from ferroptosis thereby promoting tumor progression and poor prognosis [47, 48]. Thus, our results highlight that accurate molecular subtyping of FASM in HCC patient samples is essential for formulating more effective patient treatment strategies.

Metabolic reprogramming is a hallmaker of solid tumor, which are increasingly attracting more and more attention. Aberrant FAs metabolic reprogramming based on transcriptional regulation or gene mutation has been proved to facilitate tumor growth and metastasis [49, 50]. In the tumor microenvironment, the high metabolic needs of cancer cells or decreased availability of serum-derived lipids will result in the increased FA biosynthesis. ACACA, a key regulator and an essential rate-limiting enzyme of fatty acid synthesis and oxidation process, is regarded as an attractive target for FAs metabolic diseases [51–54]. Here, the established model proved that ACACA expression was obviously increased in various cancers. The elevated expression of ACACA was relevant to worse tumor stages, grades, metastases and poor survival in many types of human tumors, especially in LIHC. Previous reports showed that the mutation of ACACA gene was associated with the survival durations in cancers [55–57]. The expression of ACACA presented a positive correlation with TMB in most cancers, including LIHC, while it was negatively correlated with BRCA and ESCA. Additionally, when we compared the relationship of ACACA and MSI, they showed a positive correlation in GBM, KIRC, LIHC, LUAD, LUSC, STAD, UCEC and CESC, and a negative correlation in with BRCA, DLBC, HNSC and THCA. These results indicated that investigating the underlying mechanisms of ACACA epigenetic modifications could help improve its clinical application in patients with various types of cancer.

Recent evidences have shown that tissue resident memory CD8^+^ T (T_RM_) cells are closely related to prognosis of cancer patients [58, 59]. However, the stromal barrier, immunosuppressive microenvironment, and insufficiency tumor-associated antigens limit the clinical efficacy of targeted T_RM_ cells in solid tumors. Our study observed that Cluster2 and low FASM-risk patients with HCC had an anti-tumor immunity with high infiltrations of NK cells and M1 macrophages, while the immunosuppressive regulatory T cells and M0 macrophages were more abundant in Cluster1 and high FASM-risk group. Notably, with respect to immunotherapy guidance, we observed enhanced immune escape potential in Cluster1 and high FASM-risk groups. Low FASM-risk patients get more satisfactory clinical benefits after immune checkpoint (PD-1/PD- L1) blockade therapies. Furthermore, the enhanced stemness of tumor cells caused by abnormal FAs metabolism reduces the survival time of patients and increases the possibility of cancer recurrence [60, 61]. Consequently, CSC niches works by recruiting immunosuppressive cells, such as cancer-associated fibroblasts, Tregs and M2 macrophages to enhance their pro-tumor activity [62]. In addition, Cluster1 had a higher stemness score and chemotherapy tolerance (e.g., Sorafenib, Cytarabine and Docetaxel) compared with Cluster2. Those finding confirmed the predictive validity of the proposed molecular subtyping and prognosis risk model. Simultaneously, further studies on interfering with FASM, enhancing immune cell penetration, and influencing inhibitory TME components will become therapeutic approaches.

Currently, scRNA-seq is a powerful tool for characterizing the basic properties of cells in solid tumors. Meanwhile, the cell subtypes in TME and cellular communication have been identified [63, 64]. In this research, we used the GSE125449 scRNA-seq dataset to assess the heterogeneity of HCC. Results identified that there was a direct and strong interaction between ACACA-highly expressed CSCs with other cell subtypes, and the CD74 and ITGB1 signaling pathways mainly regulated intercellular communication. Therefore, the full effect of cellular interactions should be synthetically considered in the research direction of HCC therapy. Additionally, we confirmed that the expression of ACACA in HCC patient, SD rat liver cancer model and cell lines presented an obviously higher expression than that in normal tissues and cell lines. Notably, in vitro cell experiments also exhibited that the proliferation, migration and stemness of HCC cell lines were greatly decreased when the ACACA was knocked down. Thus, understanding the potential metabolic changes that occur during HCC development is critical to ensuring new strategies for cancer treatment that target the specific nutrition needs of cancer cells.

Although the research findings are helpful for a more particular knowledge of the molecular characteristics, physiologic function and prognosis of FASM, they still have some limitations. First, the prognosis value of twenty-six FASM differential genes in HCC was identified, but we did not validate it in other cancers. At the same time, more in vivo studies of ACACA carcinogenesis are needed to demonstrate its prognostic value. And the exact molecular mechanism of ACACA affecting HCC progression is still unknown. Therefore, we intend to do more in-depth research in subsequent studies.

## Conclusion

Our findings examined the key role of FASM in HCC. We identified the bionomics and immunologic properties of FASM subtypes in HCC, and evaluated its clinical value. Importantly, we verified the characteristics and role of hub gene (ACACA) of FASM in HCC progression by in vivo and in vitro functional experiments. Based on the above results, FASM model has the expected clinical implications for prognostic assessment and may help physicians to formulate treatment strategies from a nutritional perspective.

### Supplementary Information

**Additional file1:** Supplementary Materials.

**Additional file 2: Table S1.** TCGA_LIHC_Clinical.

**Additional file 3: Table S2.** ICGC_LIRI_JP_Clinical.

**Additional file 4: Table S3.** Gene sets of FASM.

**Additional file 5: Table S4.** The clinical characteristics of 5 HCC samples.

## Data Availability

The datasets used and/or analysed during the current study are available from the corresponding author upon reasonable request.

## Abbreviations

HCC: Hepatocellular carcinoma
FASM: Fatty acids synthesis and metabolism
scRNA-seq: Single-cell RNA sequencing
GEO: Gene expression omnibus
TCGA: The cancer genome atlas
ICGC: International cancer genome consortium
OS: Overall survival
tSNE: T-Distributed stochastic neighborhood embedding
ssGSEA: Single-sample gene set enrichment analysis
CIBERSORT: Estimating relative subsets of RNA transcripts
KEGG: The kyoto encyclopedia of genes and genomes
OCLR: One-class logistic regression
CSCs: Cancer stem cells
LASSO: Least absolute shrinkage and selection operator
TIME: Tumor immune microenvironment
MDSCs: Myeloid-derived suppressor cells
RNA-seq: RNA sequencing
PCA: Principal component analysis
TIDE: Tumour immune dysfunction and exclusion
GDSC: Drug sensitivity in cancer
PPI: Protein protein interaction
ACACA: Acetyl-CoA carboxylase1
qRT-PCR: Quantitative real-time PCR
