# Supplementary material for: Identification of fatty acids synthesis and metabolism-related gene signature and prediction of prognostic model in hepatocellular carcinoma
Source: Cancer Cell Int. 2024 Apr 7;24:130. doi: 10.1186/s12935-024-03306-4 (PMC11000322; doi:10.1186/s12935-024-03306-4)
Supplement: Supplementary file 1 — Additional file1: Supplementary Materials. [file 12935_2024_3306_MOESM1_ESM.docx]

**Additional file information**

**Identification of fatty acids synthesis and metabolism-related gene signature and prediction of prognostic model in hepatocellular carcinoma**

Ai Zhengdong^1^, Xing Xiaoying^1^, Fu Shuhui^1^, Liang Rui^1^, Tang Zehui^1^, Song Guanbin^1^, Yang Li^1^, Tang Xi^2^**^*^**, Liu Wanqian^1^**^*^**

1. Key Laboratory of Biorheological Science and Technology (Chongqing University), Ministry of Education, College of Bioengineering, Chongqing University, Chongqing, P. R. China
2. Gastrointestinal Cancer Center, Chongqing University Cancer Hospital, Chongqing, P. R. China

**^*^**Correspondence:

Liu Wanqian, professor

Bioengineering Institute of Chongqing University, 174 Shazheng Street, Chongqing 400000, China. E-mail: [wqliu@cqu.edu.cn](mailto:wqliu@cqu.edu.cn)

Tang Xi, M.D.

Gastrointestinal Cancer Center, Chongqing University Cancer Hospital, Chongqing, 400000, China. E-mail: tangxi@cqu.edu.cn

The Additional information file contains supplementary figures (Figure S1 to S8).


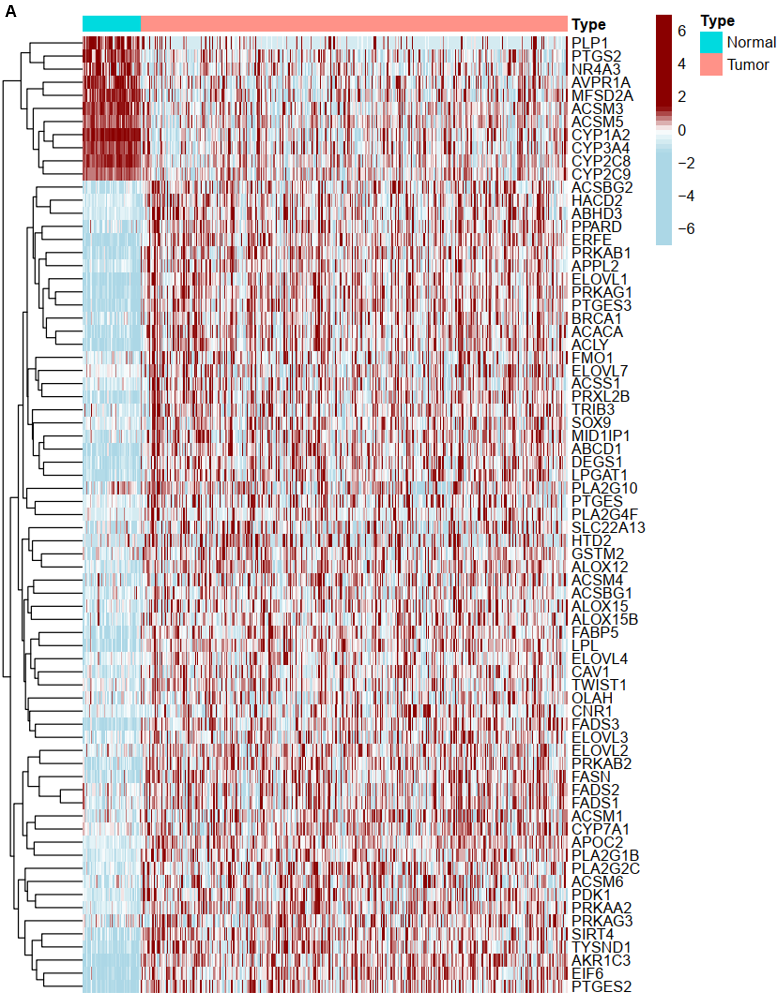


Figure S1. Heat map of differentially expressed FASM genes in HCC tumor compared to normal.


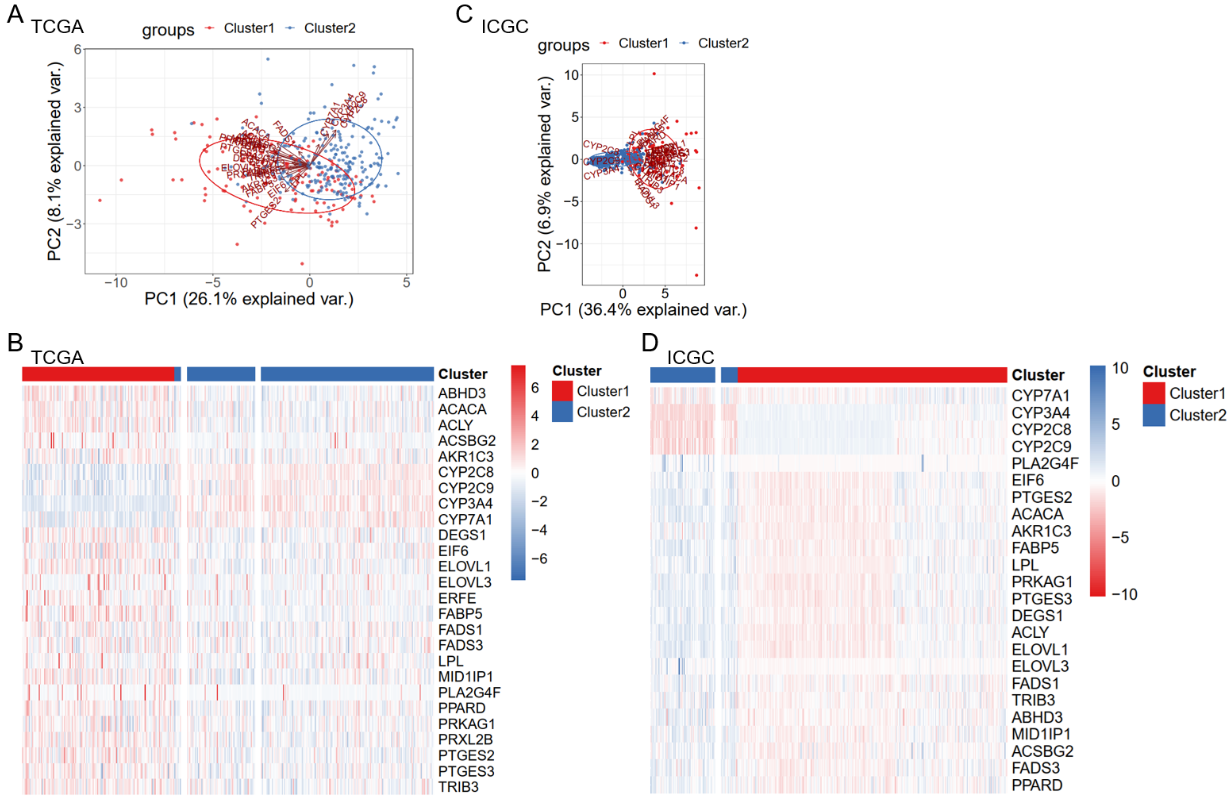


Figure S2. Differentially expressed in two patterns of FASM. (A) PCA verified the two patterns in TCGA-LIHC. (B) Heat map of differential expression of 26 FASM genes in two clusters from TCGA-LIHC. (C) PCA verified the two patterns in ICGC-LIRI. (D) Heat map of differential expression of 26 FASM genes in two clusters from ICGC-LIRI.


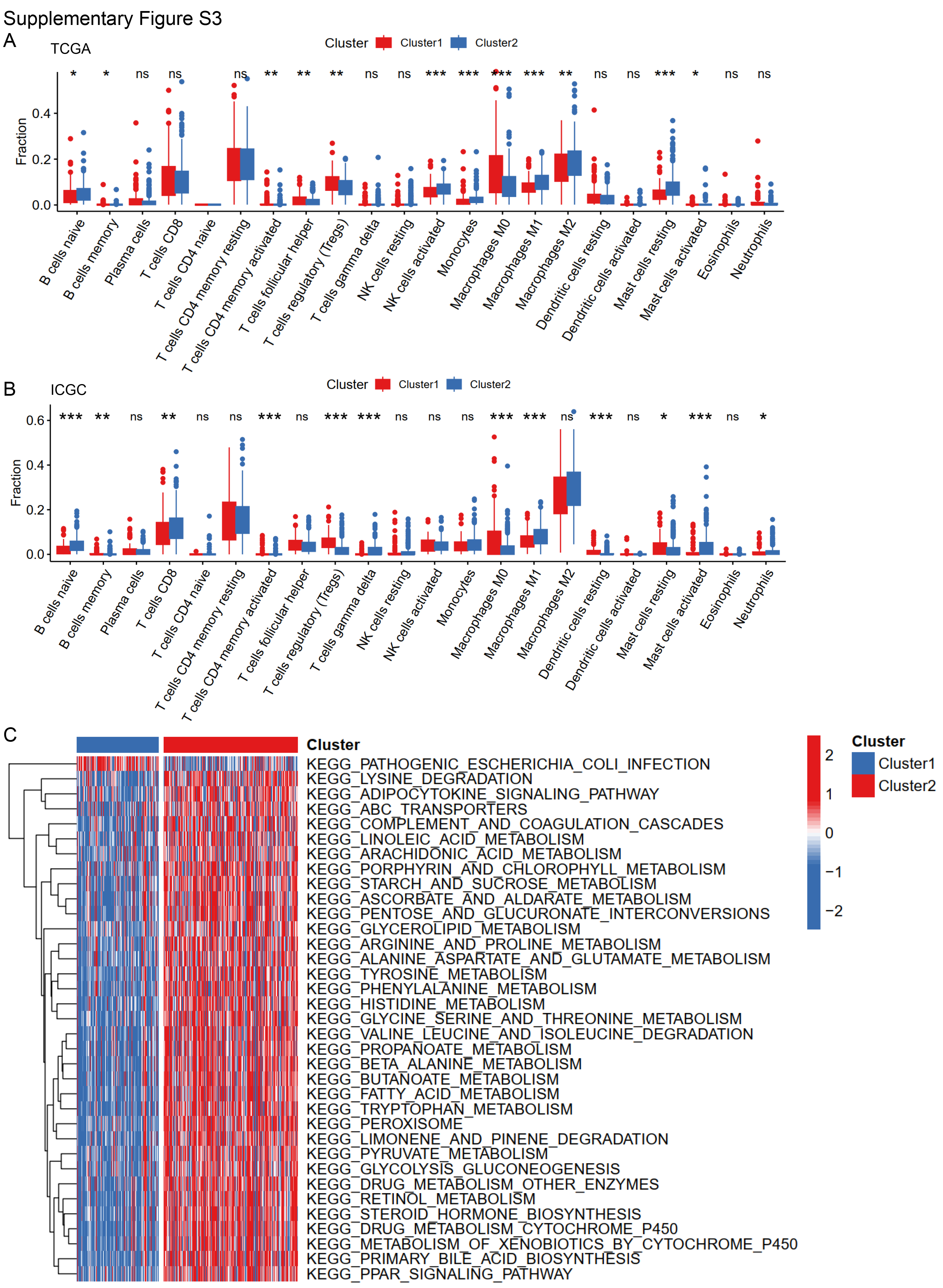


Figure S3. Correlation of FASM patterns, immune infiltrating cells and KEGG pathways. (A) Differences of the abundance of immune infiltrating cells between two FASM subtypes in TCGA. (B) Differences of the abundance of immune infiltrating cells between two FASM subtypes in ICGC. (C) Heat map of the KEGG pathways enrichment scores among FASM subtypes. *p < 0.05; **p < 0.01; ***p < 0.001; ****p < 0.0001; ns, no statistical significance.


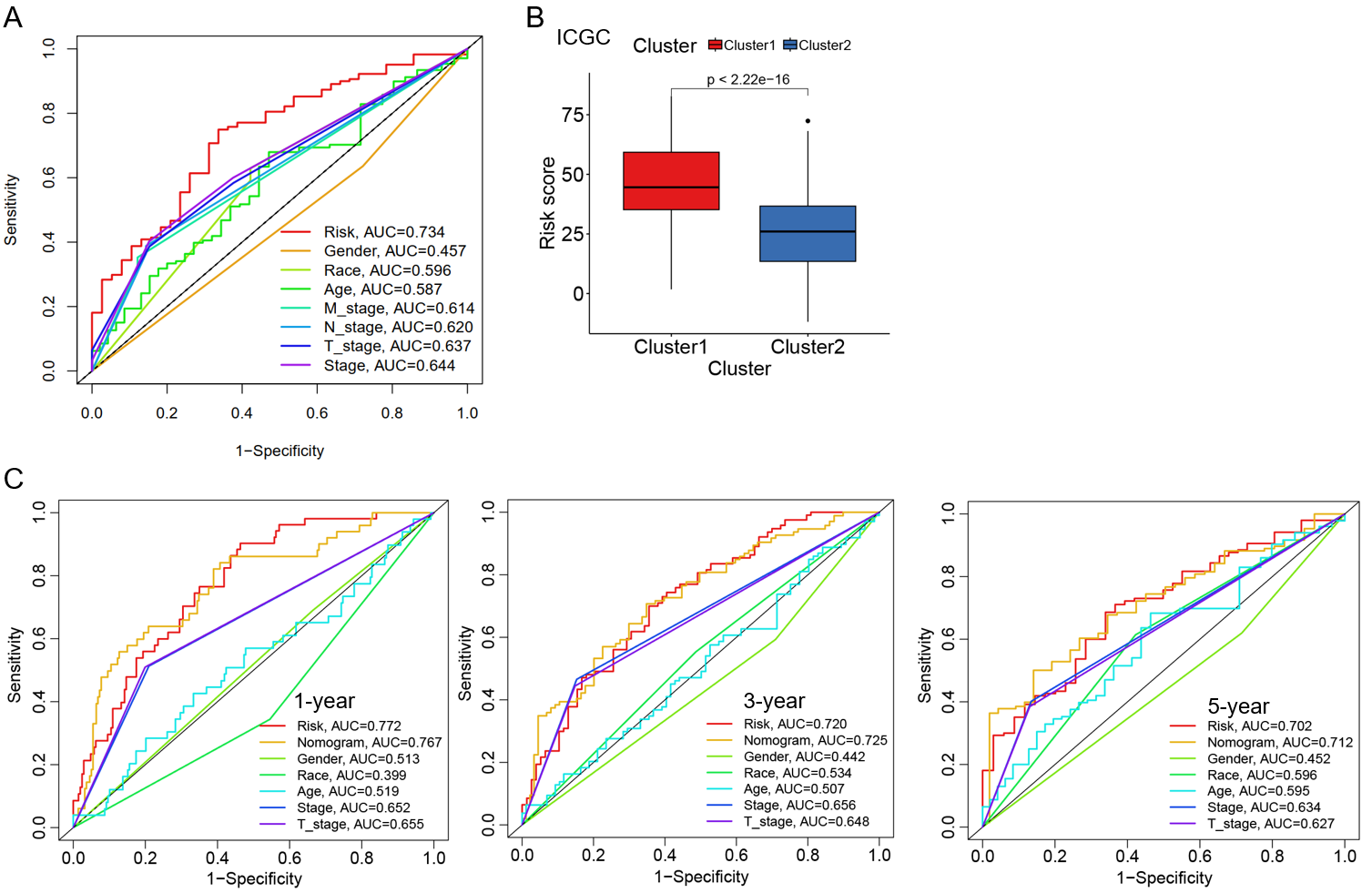


Figure S4. Verification of risk model prognostic value. (A) ROC curves for clinical parameters and risk model. (B) Correlation of risk and subtypes in ICGC. (C) ROC curves of nomograms predicting 1-year, 3-year and 5-year survival rates.


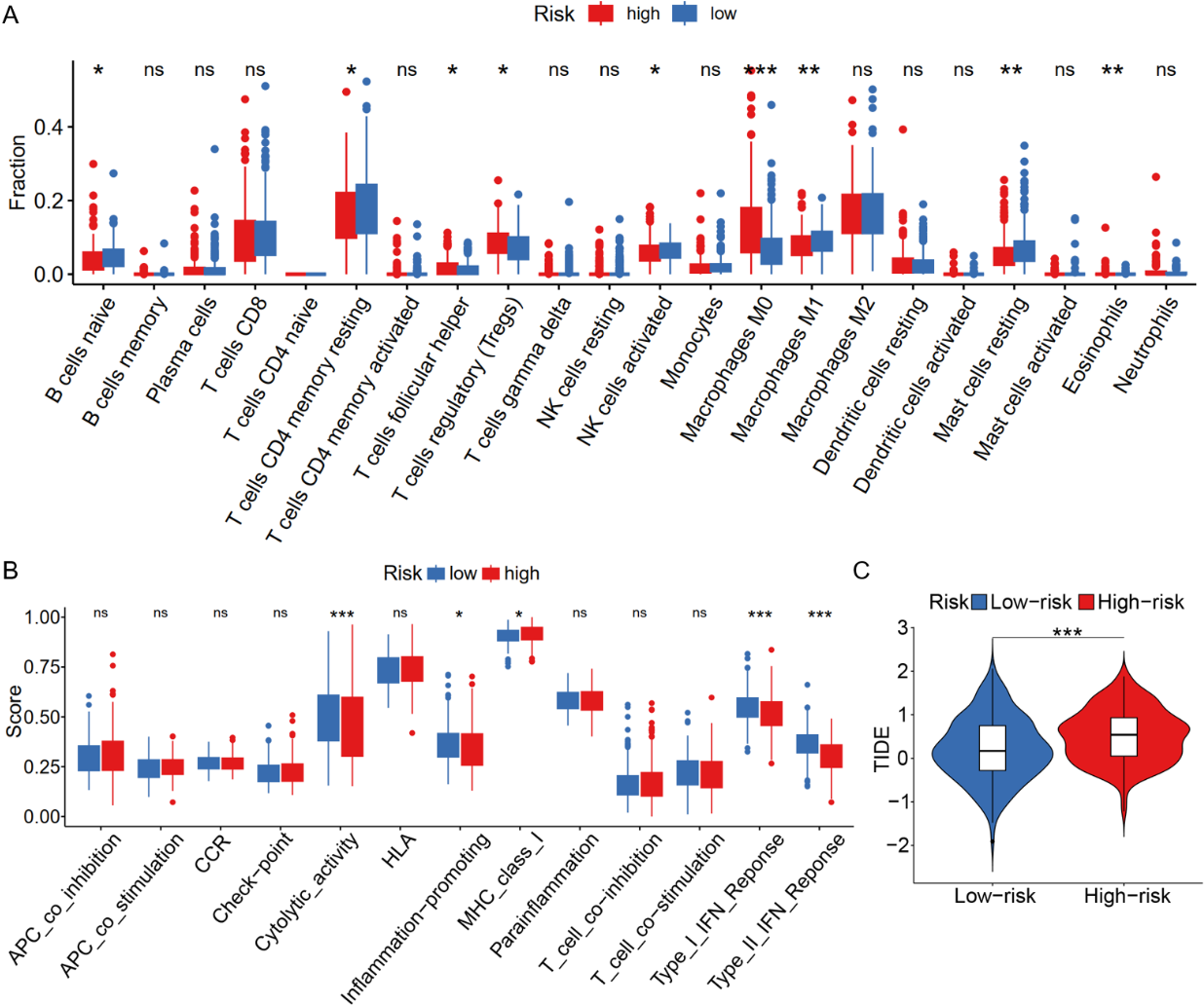


Figure S5. Correlation between FASM risk subtypes and immune infiltrating cells. (A) Differences of the abundance of immune infiltrating cells in two FASM risk subtypes. (B) Differences of the immunologic function in two FASM risk groups. (C) Difference analysis of TIDE scores in two FASM risk subtypes.


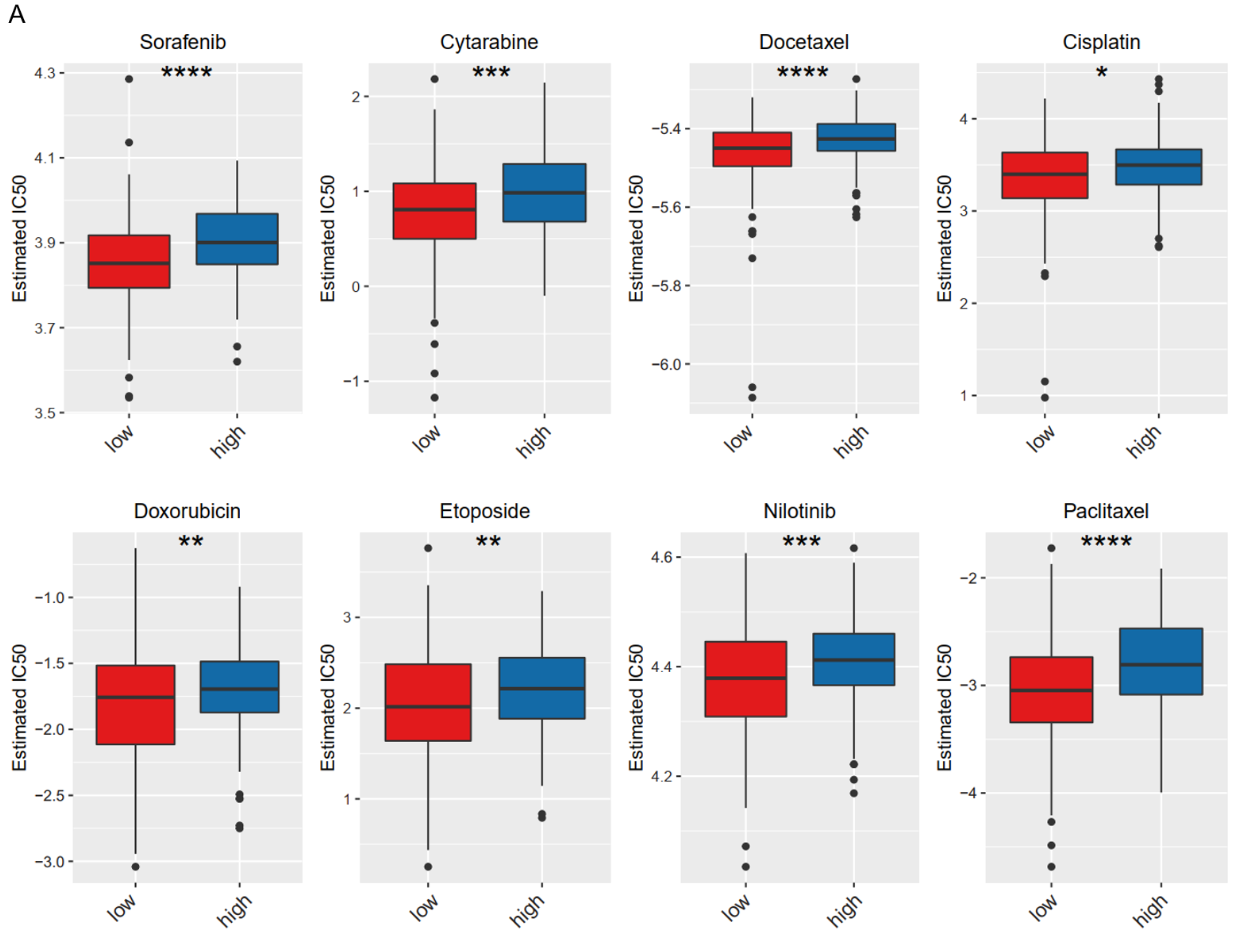


Figure S6. Response to chemotherapeutics for FASM risk subtypes in HCC. (A) Response to 8 common chemotherapeutics for risk low- and high-group in HCC. *p < 0.05; **p < 0.01; ***p < 0.001; ****p < 0.0001; ns, no statistical significance.


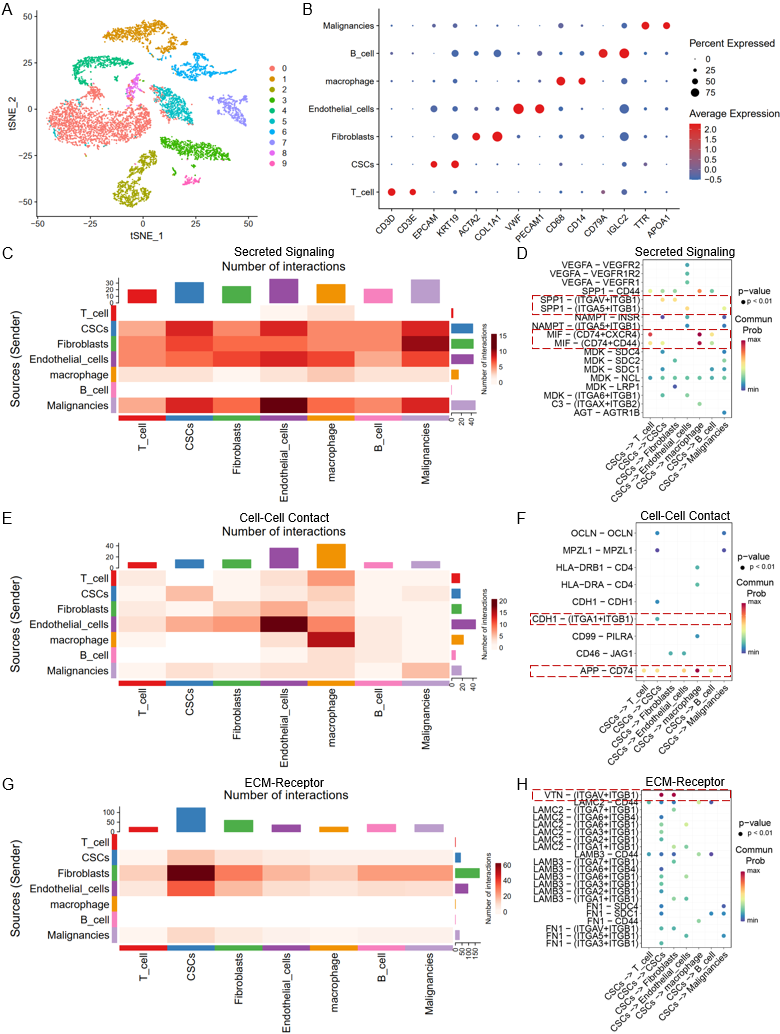


Figure S7. Cellular atlas and cell-cell communication of the HCC from GSE125449. (A) t-SNE of the 10 cell clusters. (B) Dot plot of the different cell subtypes marker genes. (C-D) Secreted Signaling-mediated interactions between CSCs and other cells. (E-F) Cell contact-mediated interactions between CSCs and other cells. (G-H) ECM-mediated interactions between CSCs and other cells.


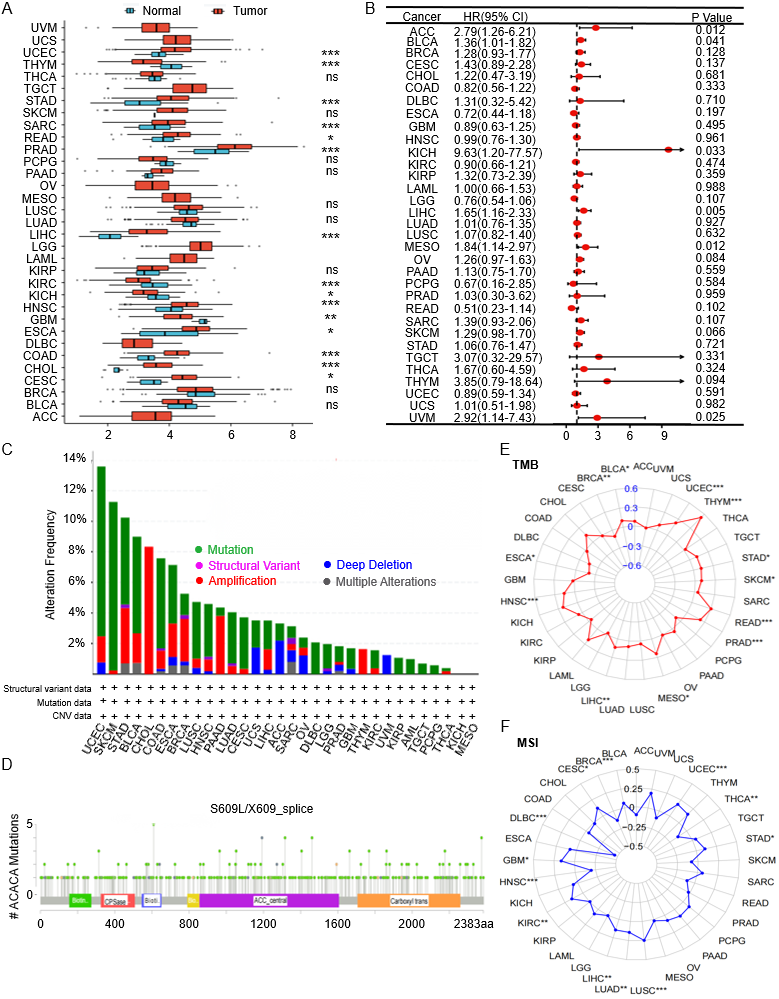


Figure S8. Molecular Characteristics of ACACA in pan-cancer. (A) The boxplot shows differences in ACACA expression levels between tumors and normal tissues. (B) The relationship between ACACA expression and overall survival in pan-cancer. (C) Mutation of ACACA in pan-cancer. (D) The mutation types, sites and frequency of ACACA genetic alteration. (E) Correlation analysis between TMB and ACACA expression in pan-cancer. (F) Correlation analysis between MSI and ACACA expression in pan-cancer. *p < 0.05; **p < 0.01; ***p < 0.001; ****p < 0.0001; ns, no statistical significance.
